# The Role of Ribonucleases and sRNAs in the Virulence of Foodborne Pathogens

**DOI:** 10.3389/fmicb.2017.00910

**Published:** 2017-05-19

**Authors:** Rute G. Matos, Jorge Casinhas, Cátia Bárria, Ricardo F. dos Santos, Inês J. Silva, Cecília M. Arraiano

**Affiliations:** Control of Gene Expression Laboratory, Instituto de Tecnologia Química e Biológica António Xavier (ITQB NOVA), Universidade NOVA de LisboaOeiras, Portugal

**Keywords:** RNA metabolism, foodborne pathogens, control of gene expression, RNase E, RNase III, PNPase, RNase R, YbeY

## Abstract

Contaminated food is the source of many severe infections in humans. Recent advances in food science have discovered new foodborne pathogens and progressed in characterizing their biology, life cycle, and infection processes. All this knowledge has been contributing to prevent food contamination, and to develop new therapeutics to treat the infections caused by these pathogens. RNA metabolism is a crucial biological process and has an enormous potential to offer new strategies to fight foodborne pathogens. In this review, we will summarize what is known about the role of bacterial ribonucleases and sRNAs in the virulence of several foodborne pathogens and how can we use that knowledge to prevent infection.

## Introduction

Globalization has important implications for food safety considering the exchanging of food around the world. Contaminated food represents a risk for human health and has an enormous negative impact in economy. Scientists are making great progresses in securing food by studying the biology, life cycles, and infection processes of foodborne pathogens.

RNA metabolism has recently been exploited for the development of new antimicrobials. Ribonucleases (RNases) are crucial enzymes for the cell, since they degrade RNA and are involved in the processing and quality control of transcripts (Arraiano et al., 2010). RNases can be divided into endoribonucleases, which cleave RNA internally, and exoribonucleases, which degrade the RNA by removing terminal nucleotides from the extremities. In the last years, the discovery of small non-coding RNAs (sRNAs) unraveled a new world of post-transcriptional regulatory networks, which cooperate with RNases in the control of gene expression. With the development of new technologies, many sRNA molecules were identified and shown to be important players in bacterial virulence.

The aim of this review is to provide a broad look to what is known about the role of bacterial ribonucleases and sRNAs in virulence of several foodborne pathogens and discuss their potential for the development of new therapeutic applications.

## Exoribonucleases

### Polynucleotide Phosphorylase (PNPase)

Polynucleotide phosphorylase (PNPase) is a phosphorolytic exoribonuclease that degrades RNA molecules from the 3′ end. However, when inorganic phosphate concentration is low, PNPase is also able to add heteropolymeric tails to the 3′ end of the RNAs. It is also involved in the exchange reaction between free phosphate and the β-phosphate of ribonucleoside diphosphates (Grunberg-Manago et al., 1955; Grunberg-Manago, 1963; Littauer and Soreq, 1982).

Polynucleotide phosphorylase is widely conserved and is not essential for survival at optimal temperatures (Donovan and Kushner, 1986). However, PNPase has been reported to be essential for adaptation and growth at low temperatures in *Escherichia coli*, *Yersinia*, *Salmonella, Staphylococcus aureus*, and *Campylobacter jejuni* (Goverde et al., 1998; Beran and Simons, 2001; Clements et al., 2002; Rosenzweig et al., 2005, 2007; Haddad et al., 2009; Bonnin and Bouloc, 2015). Refrigeration is typically used for the control of bacterial growth in food. The ability of foodborne pathogens to persist and grow at lower temperatures is a major problem for food safety and public health. The role of PNPase in adaptation to cold could be very important in the development of new strategies for food conservation.

Polynucleotide phosphorylase also seems to be directly or indirectly involved in the expression of different virulence factors in some pathogens. In *Salmonella*, PNPase activity decreases the expression of genes from the pathogenicity islands SPI-1 (containing genes for invasion) and SPI-2 (containing genes for intracellular growth) (Clements et al., 2002). In *Yersinia*, PNPase modulates the type three secretion system (T3SS) by affecting the steady-state levels of T3SS transcripts, controlling the secretion rate. This is probably the reason why PNPase deletion results in a less virulent strain in a mouse model (Rosenzweig et al., 2005, 2007). In *E. coli* O157:H7 the deletion of PNPase decreased its adhesion to epithelial cells and colonic epithelial tissues (Hu and Zhu, 2015). In *C. jejuni* it was shown that PNPase mutants have swimming limitations, colonization delay, decrease of cell adhesion/invasion ability, and reduced virulence (Haddad et al., 2012).

### RNase R

RNase R is a hydrolytic enzyme that degrades RNA processively in the 3′–5′ direction. Its activity is modulated according to growth conditions and is induced under several stress conditions, namely in cold shock (Cairrão et al., 2003; Chen and Deutscher, 2005). It has also been shown to be required for virulence in several pathogens.

In *Aeromonas hydrophila*, an emerging foodborne, RNase R was shown to be crucial for cell survival at low temperatures, and it influences the motility and virulence in mice (Erova et al., 2008). In *Shigella flexneri*, the causative agent of dysentery, RNase R is extremely important for the expression of a variety of invasion factors, and decreases expression of virulence factors, epithelial cell invasion, and hemolytic activity (Tobe et al., 1992; Cheng et al., 1998). In *C. jejuni*, RNase R was shown to be active in a wide range of conditions, which could be crucial for the adaptation during the infection process. This exoribonuclease was also shown to be involved in adhesion and invasion of eukaryotic cells (Haddad et al., 2014). In *Helicobacter pylori* this enzyme was shown to promote apoptosis of host cells while enhancing bacterial chemotaxis and motility, features that are required for optimal virulence (Tsao et al., 2009).

## Endoribonucleases

### RNase E

RNase E is essential for cell growth in all organisms where it is present. RNase E cleaves internally single-stranded RNA preferring AU-rich regions (Arraiano et al., 2010). In *Salmonella*, the major role of RNase E is to degrade mRNAs, including essential genes and genes implicated in virulence and intracellular growth (Chao et al., 2017). RNase E was shown to cleave the *mgtC* mRNA important not only for virulence in a murine model but also for survival within macrophages (Lee and Groisman, 2010). Moreover, studies using as infection model *Galleria mellonella* demonstrated that a *Salmonella* RNase E mutant is less virulent, the motility is impaired, and the proliferation inside the host is reduced (Viegas et al., 2013). Additionally, RNase E also contributes for biofilm development and antibiotic susceptibility in *Salmonella* (Saramago et al., 2014b). In *Y. pseudotuberculosis* RNase E was shown to be important for survival within macrophage-like cells and in the regulation of the T3SS (Schiano and Lathem, 2012).

### RNase III

RNase III is a hydrolytic enzyme specific for double stranded RNAs (dsRNAs). It was shown to have an important role not only in rRNA and tRNA maturation but also in mRNA decay. Although not essential, in the absence of RNase III cells show defective growth when compared to the wild-type (Arraiano et al., 2010). In *Salmonella*, RNase III has been implicated in the control of important virulence factors (Lee and Groisman, 2010). Using *G. mellonella* as a host model, a mutation in RNase III reduced the *Salmonella* virulence capacity (Viegas et al., 2013). It was also shown that RNase III contributes for biofilm development and antibiotic susceptibility in *Salmonella* (Saramago et al., 2014b).

In *S. aureus* RNase III regulates the expression of factors implicated in cell adhesion and escape to immunity by degrading sRNA/mRNA duplexes (Bonnin and Bouloc, 2015). In *C. jejuni* RNase III was shown to be active in different conditions which can be important during infection. Moreover, it was able to complement the *E. coli* RNase III mutant restoring the 30S rRNA processing and PNPase regulation (Haddad et al., 2013). Considering the important functions that PNPase play in *C. jejuni* virulence (see above), this also confers to RNase III an important role in *C. jejuni* biology.

### YbeY

YbeY is a metalloprotein important for rRNA processing, which is also involved in gene silencing (Jacob et al., 2013; Saramago et al., 2017). In *Vibrio cholerae* YbeY is essential and is involved in pathogenesis, as its depletion was shown to have a negative impact in cholera toxin production and a reduced colonization of mouse intestines (Vercruysse et al., 2014). In *Y. enterocolitica*, YbeY also has a high impact in the pathogenicity of this bacterium. In the absence of YbeY several virulence factors are differentially expressed, resulting in a drastic defect in adhesion and a decreased ability of *Y. enterocolitica* to infect eukaryotic cells (Leskinen et al., 2015).

### Other Endoribonucleases

In *Listeria monocytogenes*, the inactivation of the putative RNase HII, called Imo1273, caused a strong attenuation of virulence in a mouse model. Interestingly, overexpression of this protein also attenuates virulence, which means that Imo1273 is expressed in an appropriate level to promote an efficient infection (Bigot et al., 2009). As such, Imo1273 seems a good target to be further studied to control *L. monocytogenes* virulence.

In *S. aureus*, it was discovered an RNase Y ortholog that regulates virulence genes (Bonnin and Bouloc, 2015). It was also shown that RNase Y and PNPase act in an opposing manner to regulate *S. aureus* virulence (Numata et al., 2014).

## RNA Regulators

### Hfq

The bacterial Hfq is an RNA-chaperone critical for the sRNA-mediated regulation and it is mainly known for facilitating the imperfect base-pairing between sRNA and their targets (Vogel and Luisi, 2011; Andrade et al., 2013). Hfq has been related to pathogenesis in many microbes. Inactivation of Hfq in *Cronobacter sakazakii* leads to an attenuated virulence phenotype. *In vivo* dissemination, invasion ability and survival in host cells are negatively affected in the absence of Hfq (Kim et al., 2015). The RNA-chaperone is also important for *Y. pseudotuberculosis, Salmonella, L. monocytogenes* and *S. flexneri* virulence processes (Christiansen et al., 2004; Sharma and Payne, 2006; Sittka et al., 2007; Schiano et al., 2010) and can therefore serve as a potential therapeutic target in foodborne-related diseases.

### sRNAs

sRNAs have been extensively implicated in the adaptation of pathogenic bacteria to different environments upon entry in the host (for a thorough review see Silva et al., 2011; Ortega et al., 2014; Saramago et al., 2014a; Svensson and Sharma, 2016).

SgrS is a bifunctional sRNA expressed in several organisms. It is specifically associated with the glucose-phosphate stress preventing inhibition of cell growth (Gorke and Vogel, 2008). In *Salmonella* this sRNA also regulates the expression of the *Salmonella*-specific virulence effector SopD which is injected into the host upon infection (Brumell et al., 2003; Papenfort et al., 2012). SgrS binds directly to the early coding sequence of *sopD* mRNA leading to the translation inhibition with the concomitant degradation of the mRNA target (Papenfort et al., 2012). The deletion of this sRNA leads to an attenuation in *Salmonella* virulence in mice (Santiviago et al., 2009).

Contrarily to what happens in other pathogenic organisms, there are few sRNAs described so far in *S. flexneri*. Interestingly, all of these sRNAs are connected with virulence processes and are expressed in specific environmental conditions (for a review see, Fris and Murphy, 2016). A curious example is RnaG sRNA that is encoded in the *S. flexneri* virulence plasmid pINV. RnaG is known to downregulate the expression of the virulence gene *icsA* that encodes an outer membrane protein crucial for bacteria spreading inside the host (Bernardini et al., 1989; Giangrossi et al., 2010). RyfA1 is another sRNA that affects *S. flexneri* multiplication in the host cells. Specifically, its over-expression leads to the elimination of *ompC* mRNA that encodes the major outer membrane protein C. The dramatic disappearance of the *ompC* transcript results in an inhibition of cell-to-cell spread, a mechanism that is crucial for pathogenesis (Bernardini et al., 1993; Fris et al., 2017). Interestingly, the expression of RyfA1 was shown to be negatively regulated by other sRNA, RyfB1 (Fris et al., 2017).

The Csr system is a global regulator of bacterial central metabolism that regulates several important mechanisms including virulence (Heroven et al., 2012; Vakulskas et al., 2015). The main component of this system, CsrA, regulates the expression of several mRNAs and is regulated by two sRNAs, CsrB, and CsrC that sequester CsrA inhibiting its activity (Liu et al., 1997; Weilbacher et al., 2003). In the absence of both RNAs, *S. flexneri* was able to invade eukaryotic cells more efficiently (Gore and Payne, 2010), due to the consequent increasing of free CsrA. In *Salmonella*, both the loss and overexpression of CsrA were shown to decrease invasion (Altier et al., 2000), highlighting that the level of CsrA and its sRNAs regulators must be controlled to allow a correct invasion. In *Y. pseudotuberculosis*, these sRNAs were also associated with virulence through their role in the regulation of RovA, a global transcriptional factor (Heroven et al., 2008). There were several other riboregulators described and associated with *Yersinia* virulence processes (for a review see, Nuss et al., 2017).

Besides its role on iron metabolism, RyhB was shown to be important for *Shigella dysenteriae* virulence by inhibiting the transcription of *virB* (Murphy and Payne, 2007), a transcription factor that promotes the expression of several virulence genes and is encoded in the *S. dysenteriae* large virulence plasmid (Adler et al., 1989). The repression of *virB* using a strain overexpressing RyhB affects the invasion efficiency of eukaryotic cells, plaque formation and effector protein secretion (Murphy and Payne, 2007). The expression of the two RyhB *Salmonella* homologes, RfrA and RfrB, was shown to be increased inside fibroblasts, and in murine macrophages (Padalon-Brauch et al., 2008; Ortega et al., 2012; Leclerc et al., 2013) emphasizing their importance in *Salmonella* intracellular replication.

In *Vibrio parahaemolyticus* it was shown that the sRNA Spot 42 downregulates VP1682 – a chaperone protein of the T3SS1 – in an Hfq-dependent fashion. Deletion of Spot 42 led to an increase in cytotoxicity in Caco-2 cell cultures (Tanabe et al., 2015). In *V. cholerae*, the sRNA VrrA has major implications to bacterial fitness and virulence. VrrA regulates the expression of outer membrane porins, outer membrane vesicles (OMVs) and biofilm formation. This sRNA represses OmpA by base-pairing with the Shine-Dalgarno and coding region of the target mRNA. Downregulation of OmpA correlates with an increase of OMV, which has been associated with new pathways of toxin delivery and evasion of the host immune system (Song and Wai, 2009). Deletion of VrrA sRNA resulted in an attenuation of virulence of *V. cholerae* in a mouse model, linking this sRNA to pathogenesis (Song et al., 2014).

The pathogenicity island locus of enterocyte effacement (LEE) present in the enterohemorrhagic *E. coli* (EHEC) and enteropathogenic *E. coli* (EPEC) encodes the T3SS responsible for the delivery of effector molecules to the infected host cells. Overexpression of the Hfq-dependent sRNA350 was shown to activate expression of LEE encoded operons by regulating the master regulator *ler*. Interestingly, sRNA350 is located within LEE and originates from 3′-UTR region of the *cesF* mRNA, a chaperone necessary for EPEC virulence (Gruber and Sperandio, 2015). This sRNA does not appear to be processed and it carries out its function as part of the *cesF* transcript (Bhatt et al., 2016). Consequently, sRNA350 constitutes an example for the diverse nature of sRNA biogenesis and action.

### Others

CRISPR RNAs are long repeat sequences interspaced by non-repetitive spacer sequences that provide bacteria with an adaptive immunity against foreign genetic elements. *L. monocytogenes* possesses a cas-less CRISPR system (RliB-CRISPR) with an unusual base-pairing between the repeat sequences and the adjacent spacers (Sesto et al., 2014). RliB sRNA is important for *L. monocytogenes* virulence as it was found to be upregulated in bacteria grown in mouse intestine. Moreover, upon RliB inactivation bacterial cells accumulated much faster than the parental strain in mouse liver (Toledo-Arana et al., 2009). Bacteriophages can often impact the expression of bacterial virulence factors. Therefore, the RliB-CRISPR system can pose as an important mechanism for regulation of *L. monocytogenes* virulence by modulating the crosstalk between bacteriophages and bacteria during infection.

In the 5′ UTR of *shuA*, a virulence gene that encodes an outer-membrane heme receptor, it was discovered the first RNA thermometer described in *S. dysenteriae*. This RNA structure senses the temperature of the human body upon invasion (Mills and Payne, 1995, 1997; Kouse et al., 2013). ShuA enables the utilization of heme as a source of iron and is only expressed when iron is depleted and at temperatures corresponding to the human body (Kouse et al., 2013). In *L. monocytogenes* it was shown that the virulence regulator PrfA was controlled by a RNA thermometer located at the 5′ UTR of the *prfA* transcript. At lower temperatures, the 5′ UTR of *prfA* forms a secondary structure which results in translation inhibition. At 37°C the secondary structure is not formed, and translation occurs (Johansson et al., 2002). These are good examples of sophisticated mechanisms that efficiently activate virulence genes at 37°C.

## Conclusion

Growth of foodborne pathogens in food is mainly controlled by using refrigerated temperatures. Foodborne pathogens are able to survive at low temperatures by adapting their gene expression, which represents a challenge to developing new strategies for making food safer. The ribonucleases PNPase and RNase R are very important for the adaptation to lower temperatures by regulating cold survival transcripts and increasing their levels upon a cold shock. As such, these proteins could be very interesting in the developing of new strategies to prevent food contamination. In the last years, there was a great development in the production of polymers that can be used for food packaging, namely nanofibers that can be designed to soak certain compounds that confer antimicrobial protection (Torres-Giner, 2011). Hence, RNases offer a new perspective for developing antimicrobials that could be used in combination with such polymers. Although the discovery of inhibiting compounds is still a challenge, the use of small molecules that target RNase E in *E. coli* was already described (Kime et al., 2015). Moreover, in *S. aureus*, it was successfully identified and validated an inhibitor of RnpA, the protein component of RNase P (Olson et al., 2011; Eidem et al., 2015).

Ribonucleases often cooperate with sRNAs in the regulation of key cellular processes, namely, in the adaptation to different extracellular environments and in the regulation of virulence factors of foodborne pathogens. By providing knowledge on the sRNA-mediated virulence and adaptation networks we are widening the scope in the look for new antimicrobial targets. Another hypothesis is the use of antisense RNAs as they were shown to inhibit growth of *E. coli*, *S. enterica*, and *S aureus* by targeting essential genes (revised in Thomason and Storz, 2010). Antisense RNAs detain the advantage of being very specific and can affect multiple targets. However, there are many variables that still need to be tested before its use.

The knowledge that we have on RNA regulators and their involvement in pathogenesis is still the tip of the iceberg. In this review, we covered only a few examples of what is known in the field. In addition, with the constant development of new tools many important players in virulence will be certainly identified. These findings will certainly be exploited in the near future to pursue good antimicrobial candidates, opening a new world of possibilities.

## Author Contributions

RGM, JC, CB, RFdS, IJS, and CMA wrote the manuscript, RGM and CMA revised and coordinated the work.

## Conflict of Interest Statement

The authors declare that the research was conducted in the absence of any commercial or financial relationships that could be construed as a potential conflict of interest.
